# Unilateral pulmonary vein atresia

**DOI:** 10.5588/ijtldopen.24.0631

**Published:** 2025-04-09

**Authors:** Y. Wang, Y. Tang, L. Sun, X. Zhang, Y. Liu, S. Xu, Y. Su, L. Zhang, X. Tang, H. Yang, Y. Shen

**Affiliations:** ^1^ Respiratory Department, Children’s Hospital Affiliated to Zhengzhou University, Henan Children’s Hospital, Zhengzhou Children’s Hospital, Zhengzhou, China;; ^2^ Radiology Department, Children’s Hospital Affiliated to Zhengzhou University, Henan Children’s Hospital, Zhengzhou Children’s Hospital, Zhengzhou, China;; ^3^ Respiratory Department II, National Clinical Research Center for Respiratory Diseases, Beijing Children’s Hospital, Capital Medical University, National Center for Children’s Health, Beijing, China;; ^4^ Department of Respiratory Diseases, Pediatric Research Institute of Xinjang Uygur Autonomous Region, Children's Hospital of Xinjang Uygur Autonomous Region, Xinjiang Hospital of Beijing Children’s Hospital, The Seventh People's Hospital of Xinjiang Uygur Autonomous Region, Urumqi, China.

**Keywords:** rare lung disease, interstitial lung disease, haemoptysis, Chinese

## Abstract

**OBJECTIVE:**

This study aims to summarise the clinical characteristics of unilateral pulmonary vein atresia (UPVA) and compare the differences between Chinese cases and all published cases worldwide.

**METHODS:**

We retrospectively enrolled 6 Chinese children with UPVA from January 2014 to January 2024 at a single centre. We reviewed their demographic data, clinical symptoms, laboratory tests, imaging examinations, treatment and prognosis to describe their clinical features. Additionally, the remaining 79 confirmed patients with UPVA, as described in 52 references, were also summarised.

**RESULTS:**

UPVA is sporadically distributed worldwide, with the highest number of reported cases in China (27/85) and the United States (20/85). In the overall cohort (85 cases), the patient median age at diagnosis was 5.2 years. The male-to-female ratio was 1:1. Right-sided UPVA was slightly more common, with a right-to-left ratio of 1.4:1. The most frequently reported clinical manifestations were recurrent pneumonia (79.2%), followed by recurrent haemoptysis (48.1%) and exercise intolerance (35.1%). Additionally, 10.4% of patients were asymptomatic. Congenital heart disease was observed in 34.1% of cases, and 20% of patients had comorbid pulmonary hypertension. The overall mortality rate was 8.9%.

**CONCLUSION:**

There were no statistically significant differences in the clinical characteristics of UPVA between Chinese patients and all published cases worldwide.

Pulmonary vein atresia (PVA) is a rare congenital malformation due to failure of incorporation of the common pulmonary vein into the left atrium.^1^ It can cause progressive pulmonary arterial hypertension (PAH) and heart failure.^2^ PVA can be classified into three types based on the extent of involvement and the stage of normal development of pulmonary venous drainage: common, unilateral, and individual PVA.^3^ Unilateral pulmonary vein atresia (UPVA) is an even rarer condition that involves the complete absence of pulmonary veins on one side of the lung. UPVA is usually diagnosed in childhood, and about half of the patients have associated congenital heart disease (CHD), such as patent ductus arteriosus (PDA), atrial septal defect (ASD), and ventricular septal defect (VSD).^4^ Herein, we present six rare cases of UPVA in Chinese children and review an additional 79 confirmed cases reported worldwide between January 1975 and January 2024. To the best of our knowledge, this represents the most extensive and comprehensive investigation into the clinical characteristics of UPVA to date.

## METHODS

Children referred to the Respiratory Department of Children’s Hospital Affiliated to Zhengzhou University from January 2014 to January 2024 were enrolled in the study after meeting the diagnostic criteria of UPVA. UPVA was diagnosed using a combination of imaging techniques, including cardiac catheterisation (CC), computed tomography angiography (CTA), digital subtraction angiography (DSA), echocardiography, and cardiac magnetic resonance imaging. Among these, CC is considered the gold standard for diagnosis. Clinical data were collected from all enrolled patients. This included demographic information, main symptoms, associated cardiac diseases, diagnostic methods, therapy and prognosis outcomes. Post-discharge follow-up occurred monthly. This study protocol was approved by the Ethics Committees of Children’s Hospital Affiliated to Zhengzhou University, China (Approval no. 2023-K-179). In addition, we conducted a comprehensive review of all the published UPVA cases worldwide by searching China National Knowledge Infrastructure, Wanfang, PubMed, EMBASE, Cochrane Library, OVID medicine and SinoMed from January 1975 to January 2024. The search strategy included the following term keys: (‘Unilateral pulmonary vein atresia’) AND (‘Pulmonary vein atresia’). Study types included clinical trials, meta-analyses, randomised controlled trials, case reports, case series or reviews. Original articles were included if they met the criteria. Duplicate reports were excluded from our final analysis. Statistical analyses were performed using SPSS software v22.0 (SPSS, Armonk, NY, USA). The data were expressed as mean ± standard deviation (SD) or median (interquartile range [IQR]) according to the distribution unless otherwise specified. The independent sample *t*-test was used for continuous variables, and the χ^2^ test (Yates correction) was used for categorical characteristics. If the data could not be transformed to approach normal distribution, a Mann–Whitney *U*-test was applied. *P* < 0.05 (two-tailed) was considered statistically significant.

## RESULTS

### Demographic and clinical data of our cohort

A total of 6 patients (2 males and 4 females) who met the inclusion criteria were recruited for this study. All patients were of Chinese origin, with no family history of intermarriage with Caucasians. The mean age at UPVA diagnosis was 4.7 (± SD3.4) years. The time from symptom onset to diagnosis ranged from 5 months to 3 years and 3 months. All 6 patients experienced recurrent pneumonia, three had recurrent haemoptysis, and three exhibited exercise intolerance. Two patients had cardiac abnormalities: one with a congenital membranous ventricular septal aneurysm (MVSA) and one with complex CHD, including atrioventricular septal defect (AVSD), complete endocardial cushion defect (CECD), pulmonary artery stenosis (PAS), double-outlet right ventricle, and PAH. Two cases involved left-sided UPVA, while four involved right-sided UPVA. All six cases were diagnosed via CTA. Among them, three patients with recurrent haemoptysis were also diagnosed through DSA and underwent interventional embolisation. Two of these patients showed improved haemoptysis post-procedure, while one continued to experience severe haemoptysis and recurrent respiratory infections, ultimately requiring right lung resection. All patients were followed up monthly post-discharge, with follow-up durations ranging from 1 year and 3 months to 5 years and 3 months. Five patients showed symptom improvement, while one was lost to follow-up.

### Review of the literature

Based on the literature review, an additional 79 patients were included (Supplementary Table S1). The distribution of all 85 cases (including the current study) is as follows: Mainland China (27 cases), United States (20 cases), Korea (8 cases), India and Spain (5 cases each), Japan, Turkey, and Canada (3 cases each), Italy, Ireland, and Brazil (2 cases each), and Germany, Lithuania, Malaysia, Vietnam, and Ethiopia (1 case each). UPVA is sporadically distributed worldwide, with the highest number of reported cases in China (27/85) and the United States (20/85). In the overall cohort (85 cases), patient median age at diagnosis was 5.2 years (range: 1 month–62 years). The male-to-female ratio was 1:1. Right-sided UPVA was slightly more common than left-sided, with a right-to-left ratio of 1.4:1. The most frequently reported clinical manifestations were recurrent pneumonia (79.2%), followed by recurrent haemoptysis (48.1%) and exercise intolerance (35.1%). Additionally, 10.4% of patients were asymptomatic. There were no significant statistical differences between the demographics, clinical symptoms, and common affected side of UPVA in Chinese patients compared to the overall population. CHD was present in 34.1% of all reported cases, with the most common types were PAH and PDA, occurring in respectively 20.0% and 9.4% of cases. The proportion of UPVA cases with CHD in China was slightly higher than the overall population, reaching 44.4%. The most common types of CHD in Chinese patients were PAH (33.3%) and ASD (14.8%). Conservative treatment was administered to 66.7% of patients, while 33.3% received surgical treatment. Symptoms improved in 69.6% of patients, while 10.7% experienced worsening symptoms. The overall mortality rate was 8.9%. There were no significant statistical differences between the treatment choices and prognosis in Chinese patients compared to the overall population (Supplementary Table S2).

### Case No. 81

**Figure 1.**
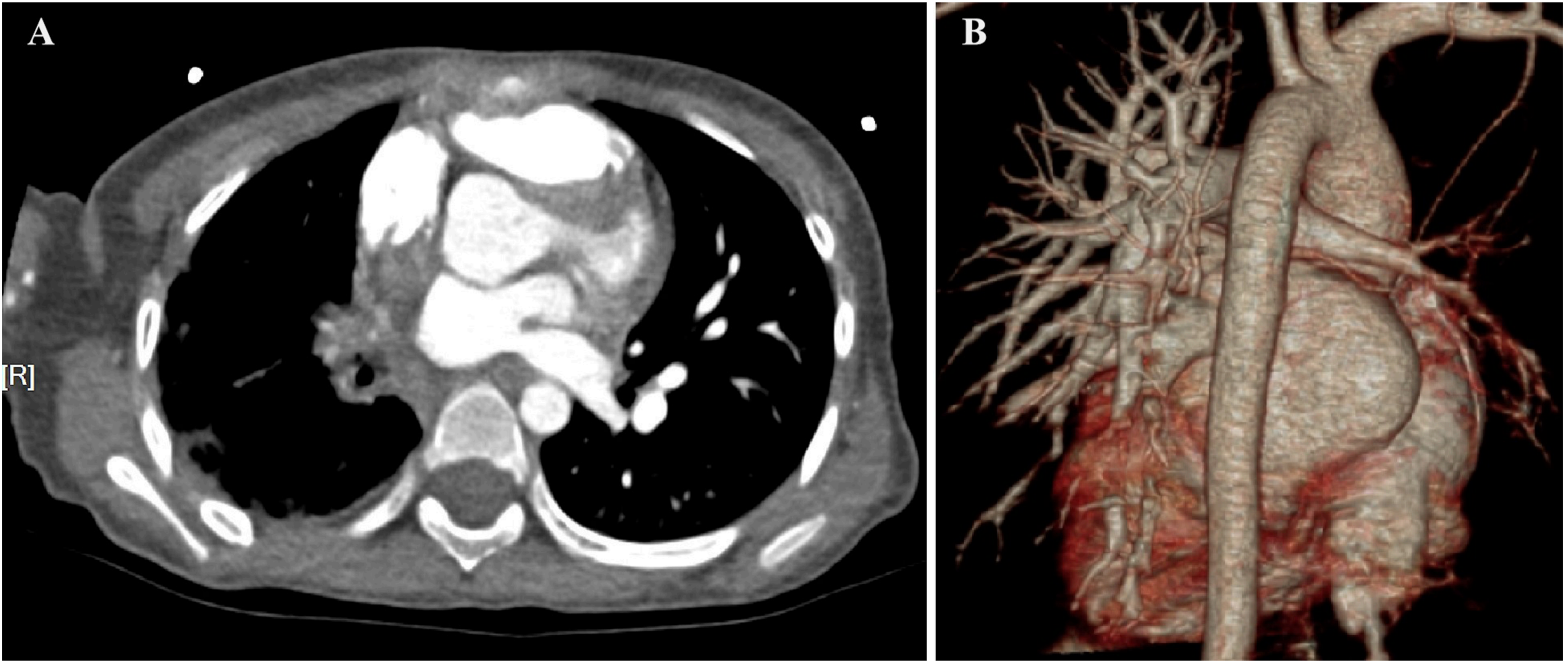
**A)** Cardiac CTA (mediastinum window) and **B)** three-dimensional vascular reconstruction of a 4-year and 11-month-old girl at her first admission (Case No. 81), both showing the absence of the right pulmonary veins, suggesting right pulmonary vein atresia. CTA = computed tomography angiography.

**Figure 2.**
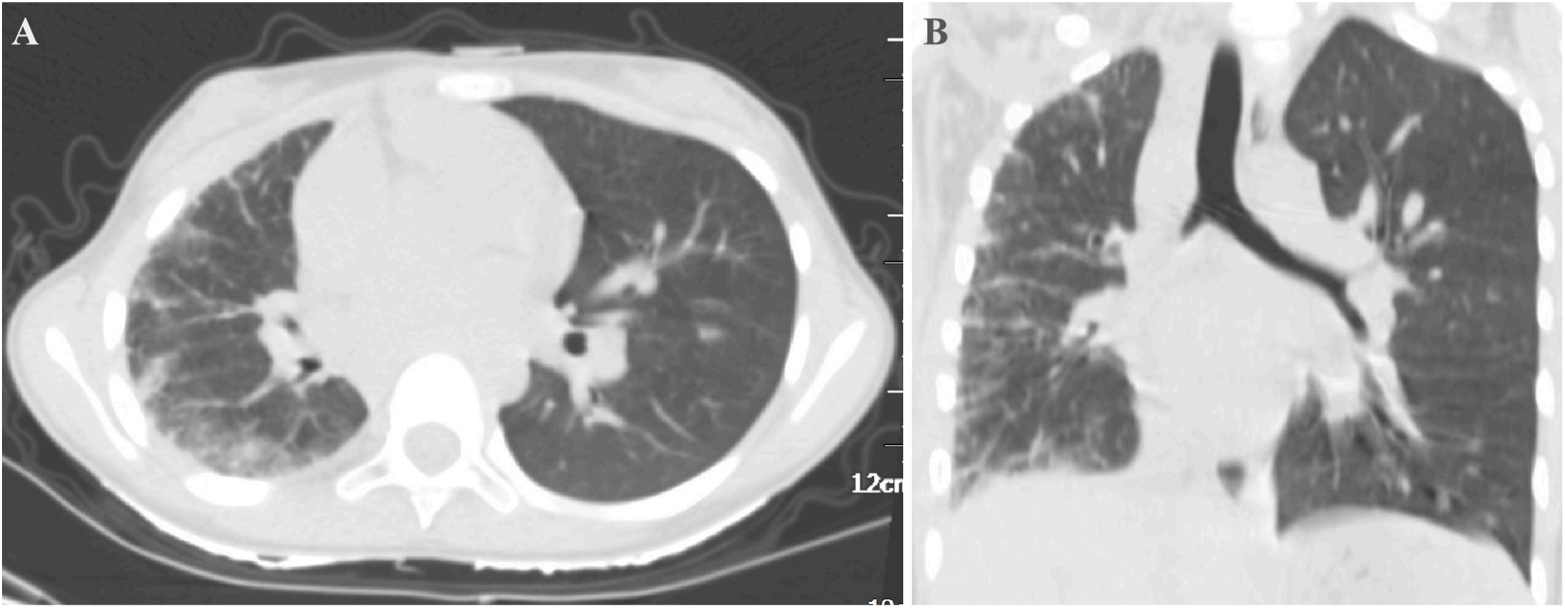
**A)** Chest CT scan (transverse view) and **B)** chest CT scan (coronal view) of Case No. 81 after 5 years and 4 months of follow-up, showing hypoplasia of the right lung, and increased interstitial lesions with irregular pleural thickening. CT = computed tomography.

A 4-year and 11-month-old girl was admitted with a 2-day history of cough and three episodes of haemoptysis, each involving 5–10 ml of fresh blood. She had no history of fever, oedema, or palpitations. Contrast-enhanced chest CT (CECT) showed interstitial changes and reduced vascular shadow in the right lung. Echocardiography revealed hypoplasia of the right pulmonary artery and vein. Cardiac CTA showed an absence of the right pulmonary veins (Figure 1), hypoplastic right pulmonary artery and branches, aortic and left pulmonary artery dilatation, and a vascular cluster at the right hilum. UPVA combined with right bronchial artery–pulmonary artery fistula (BPF) was confirmed using DSA, and interventional embolisation was performed. Post-procedure, she had two episodes of haemoptysis, including one massive episode with 800 ml of fresh blood, managed with hemostasis and transfusion. Over a follow-up period of 5 years and 4 months, she experienced recurrent pneumonia, requiring hospitalisation 3–5 times per year, with occasional blood-streaked sputum. Currently, at 10 years and 3 months old, she is 150 cm tall (90^th^ percentile) and weighs 28 kg (10^th^–25^th^ percentile). Repeat chest CT showed hypoplasia of the right lung, and increased interstitial lesions with irregular pleural thickening (Figure 2; Supplementary Table S1).

### Case No. 82

**Figure 3.**
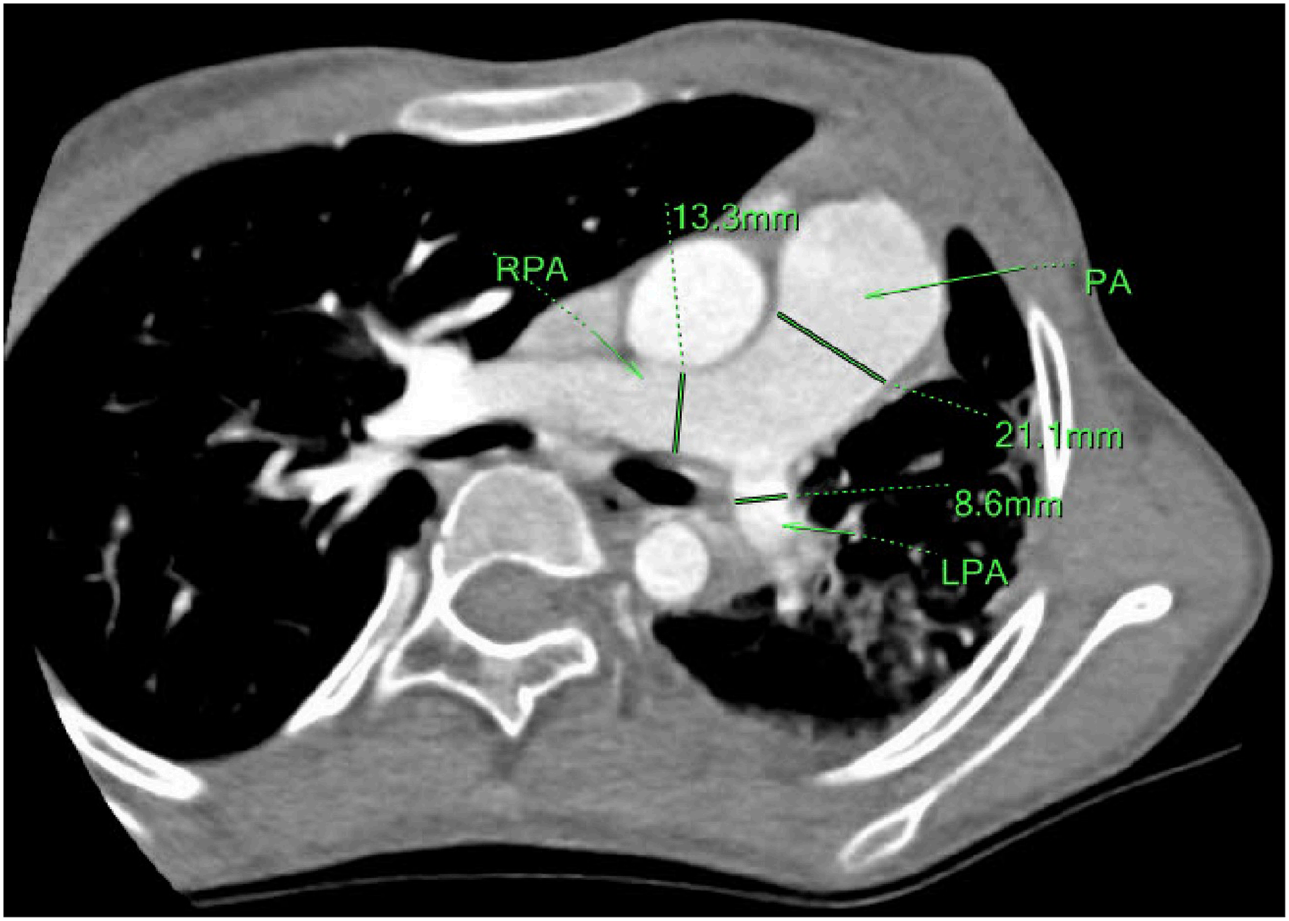
Cardiac CTA (mediastinum window) of an 11-year-old girl at her first admission (Case No. 82), showing hypoplasia of the left lung, thinning of the left pulmonary artery, and dilation of the main and right pulmonary arteries. RPA = right pulmonary artery; PA = pulmonary artery; LPA = left pulmonary artery; CTA = computed tomography angiography.

An 11-year-old girl presented with a 5-day history of cough and three episodes of haemoptysis, each involving 5–10 ml of fresh blood. She had no fever, dyspnea, or respiratory distress. Laboratory tests excluded coagulation disorders, TB, and fungal infections. Chest CT revealed reduced left lung volume with interstitial changes. Echocardiography showed an MVSA measuring 14.9 mm × 10.5 mm, with mild mitral and aortic regurgitation and a hypoplastic left pulmonary artery. There was no evidence of shunt, right ventricular hypertrophy, PAH, or aortic insufficiency. Cardiac CTA showed the absence of the left pulmonary vein, thinning of the left pulmonary artery, and dilation of the main and right pulmonary arteries (Figure 3). DSA confirmed UPVA. She has been followed up for 1 year and 4 months without recurrence of pneumonia, haemoptysis, or exertional dyspnoea. Currently, at 12 years and 4 months old, she is 150 cm tall (25^th^–50^th^ percentile) and weighs 30 kg (3^rd^ percentile) (Supplementary Table S1).

### Case No. 83

A 3-year-old girl was admitted with congenital cyanosis, recurrent pneumonia, and one episode of syncope. She had no history of haemoptysis. On examination, she exhibited cyanosis, rightward cardiac apex displacement, precordial thrill, and a coarse systolic murmur with an accentuated P2. Clubbing of the fingers and toes was noted, with peripheral oxygen saturation at 71%. Echocardiography revealed complex congenital heart disease: complete atrioventricular septal defect, double outlet right ventricle, pulmonary stenosis, and PAH. Cardiac CTA confirmed the diagnosis and showed anomalous drainage of the right pulmonary vein (supracardiac type), absence of the left pulmonary vein, stenosis of the left pulmonary artery, and marked enlargement of the right atrium. On Day 11 of hospitalisation, she underwent bidirectional Glenn shunt, atrioventricular valve repair, and pulmonary artery ligation with hypothermic cardiopulmonary bypass. Postoperatively, her oxygen saturation ranged from 75% to 85% on nasal cannula oxygenation. She remained tachypnoeic and cyanotic. She received meropenem and linezolid for infection control and bosentan and sildenafil to reduce PAH, along with supportive treatment for cardiac function and diuresis. After 38 days, her clinical condition and oxygen dependence gradually improved. She was discharged but was lost to follow-up (Supplementary Table S1).

### Case No. 84

A 1-year-old boy was hospitalised with recurrent pneumonia and three episodes of haemoptysis. The first episode involved 5 ml of fresh blood, while the subsequent episodes involved dark brown blood. He experienced dyspnoea and tachypnoea during haemoptysis. After admission, he had two more episodes of haemoptysis on Days 2 and 17, each with dyspnoea. Echocardiography was unremarkable. Chest CT showed ground-glass opacities in both lungs, suggestive of pulmonary haemorrhage and interstitial emphysema in the right lung. Pulmonary CTA showed an absence of the right pulmonary vein. He received a transfusion due to a haemoglobin level of 71 g/L, along with supportive and hemostatic therapy. Post-discharge, he continued to experience recurrent pneumonia and haemoptysis during crying episodes, with volumes ranging from 5 ml to 80 ml. At 13 months, he underwent embolisation therapy under DSA. Despite this, he continued to have recurrent haemoptysis, leading to a right pneumonectomy at 18 months. He has been followed up for 4 years and 3 months with no further haemoptysis or recurrent pneumonia. Currently, at 5 years and 3 months old, he is 110 cm tall (25^th^ percentile) and weighs 15 kg (below the 3^rd^ percentile) (Supplementary Table S1).

## DISCUSSION

UPVA is an extremely rare congenital malformation, with its incidence remaining unknown. Currently, 79 cases of this disease have been reported worldwide from January 1975 to January 2024. There are no clear racial or regional differences in the incidence of this disease, with reported ages of onset ranging from 1 month to 62 years. Most cases are sporadic reports, with only one report of a family among African immigrants.^5^ China is the world’s most populous country, with a population of approximately 1.4 billion. However, to date, only 27 UPVA patients of Chinese origin have been reported in the literature, with most publications being case reports and no epidemiological data on prevalence available. To note, most cases have been diagnosed in the past 5 years, suggesting that the actual incidence of UPVA in China may be significantly underestimated. Contributing factors may include insufficient awareness of the disease, misdiagnosis, underdiagnosis, and under-reporting.

UPVA has an insidious onset, with heterogeneous clinical features. It is often misdiagnosed as TB, leading to delayed treatment.^6–9^ By summarising all reported cases of UPVA, we found that the most frequently reported clinical manifestations were recurrent pneumonia (79.2%), followed by recurrent haemoptysis (48.1%) and exercise intolerance (35.1%). Additionally, 10.4% of patients were asymptomatic. Previous reports indicated that approximately 50% of UPVA cases were associated with CHD.^10^ However, our summary of all reported cases found that CHD was present in only 34.1% of cases, with the most common types being PDA and VSD, each accounting for 9.4%. Furthermore, 20% of UPVA patients had comorbid PAH, which could exist independently or concurrently with CHD.

Diagnosing UPVA using imaging techniques can be challenging. Typical findings on chest X-ray scans include underdeveloped lungs on the affected side, volume shrinkage, and interstitial lesions.^11–13^ These features may be related to obstruction of pulmonary venous return, and dilation of pulmonary lymphatic vessels and bronchial veins.^14,15^ Blood vessels on the unaffected side are often thicker and more numerous due to compensation.^16^ Echocardiography may be insufficient to detail the anatomical structure of the pulmonary veins. Additionally, the quality of the images and diagnostic accuracy largely depend on the operator’s experience and skill level. Cardiac CTA can diagnose approximately 85% of cases, with typical features, including no display of pulmonary veins on the affected side, underdeveloped pulmonary arteries, and compensatory vascular masses visible at the hilum of the affected side.^17^ While cardiac catheterisation remains the gold standard for haemodynamic assessment, CTA is often preferred for its detailed anatomical visualisation and non-invasive nature.^18^

There are also reports of UPVA being diagnosed in adulthood or even in old age. Most of these patients had symptoms of recurrent lung infections and/or haemoptysis during childhood, but these symptoms were not taken seriously until they gradually developed shortness of breath and progressive difficulty breathing, leading to further diagnosis. Some patients were misdiagnosed with TB during childhood and received ineffective treatment for a long time before being correctly diagnosed. There are also a few cases where symptoms of the disease began only in adulthood. For example, a Chinese female was diagnosed with UPVA at age 30 after developing dyspnoea, despite having no symptoms during childhood.^9^ Similarly, a Korean female was diagnosed at age 62 after experiencing dyspnoea since age 20 and haemoptysis at 57.^19^ Some adult patients were completely asymptomatic at diagnosis. Their pulmonary interstitial changes were usually discovered incidentally during routine check-ups or when they sought medical attention for other diseases.^7,14^

UPVA treatment depends on the patient’s symptoms and the severity of the disease. Conservative treatment is preferred for asymptomatic or mildly symptomatic patients.^20^ For patients with refractory symptoms or those who are not candidates for surgery, interventions to block collateral circulation and reduce pulmonary artery pressure may be considered.^21^ These can decrease the incidence of lung infections and alleviate haemoptysis symptoms. For patients with life-threatening haemoptysis, pneumonectomy is the preferred treatment. Lung transplantation is the ultimate cure for UPVA.^22^ Dixit et al. reported that the mortality rate of UPVA is high, with only a 49% 3-year survival rate for untreated patients.^23^ However, based on our summary of all reported cases, symptoms improved in 69.6% of patients, while 10.7% experienced worsening symptoms. The overall mortality rate was 8.9%.

In summary, we report six rare cases of UPVA in Chinese children. Meanwhile, the remaining 79 confirmed patients with UPVA described in 52 references were also summarised. There were no statistically significant differences in the clinical characteristics of UPVA between Chinese patients and all published cases worldwide. We suggest that patients with recurrent lung infections, unexplained haemoptysis, dyspnoea, or decreased exercise tolerance should be screened for UPVA. Early diagnosis and regular follow-up can control symptoms, reduce hospitalisations, and lower the risk of death, which are key to improving prognosis.

## Supplementary Material


