# Supplementary material for: Unilateral pulmonary vein atresia
Source: IJTLD Open. 2025 Apr 9;2(4):224–9. doi: 10.5588/ijtldopen.24.0631 (PMC11984521; doi:10.5588/ijtldopen.24.0631)
Supplement: Supplementary file 1 [file ijtldopen24-0631_supplementarydata1.pdf]

http://dx.doi.org/10.5588/ijtldopen.24.0631

Unilateral pulmonary vein atresia

SUPPLEMENTARY DATA

Supplementary Table S1: Cases of unilateral pulmonary vein atresia reported in the literature.

Case	References	Area	Age, y	Sex	Side	Main symptoms	Cardiac diseases	Diagnostic ways	Therapy	Outcomes
1	Nasrallah et al, 1975	USA	1.3	M	R	1	None	ECHO/DSA	S	I
2	Swischuk et al, 1980	USA	2.5	M	L	1/2	None	CC/PA	S	I
3	Swischuk et al, 1980	USA	2.0	F	R	1/2	None	CC/PA	S	NA
4	Swischuk et al, 1980	USA	2.5	M	L	1/2	None	CC/PA	NA	NA
5	Beerman et al, 1983	USA	10.0	F	R	1	None	CC	C	I
6	Beerman et al, 1983	USA	4.0	M	L	3	PDA/VSD	CC	S	I
7	Beerman et al, 1983	USA	6.5	M	L	2	PDA/PH	CC/PA	S	I
8	Cabrera et al, 1985	Spain	0.4	F	L	1	None	CC/PA/VQ scan	C	I
9	Cabrera et al, 1985	Spain	0.4	M	L	1	None	CC/PA/VQ scan	C	D
10	Cabrera et al, 1985	Spain	0.3	M	L	1/3	None	CC/PA/VQ scan	C	D
11	Cabrera et al, 1985	Spain	4	M	L	1/2	None	CC/PA/VQ scan	C	I

12	Okabayashi et al,1989	Japan	1.7	F	R	NA	PDA/VSD/PH	CSE/DSA	C	NA
13	Cullen et al, 1990	Ireland	1.0	M	L	1/2	None	CC/VQ scan	C	D
14	Cullen et al, 1990	Ireland	7.0	M	R	1	None	CC/VQ scan	C	I
15	Kim et al, 1993	Korea	5.0	F	R	1/2	PH	DSA/VQ scan	S	NA
16	Kim et al, 1993	Korea	1.0	F	R	1/2	PDA/PH	DSA	C	NA
17	Kim et al, 1993	Korea	9.0	M	R	1/2	None	DSA/MRI/VQ scan	C	NA
18	Kim et al, 1993	Korea	5.0	F	R	1/2	PH	DSA/MRI/VQ scan	S	NA
19	Kim et al, 1993	Korea	0.1	F	R	3	ASD/VSD/PDA/PH	DSA	C	NA
20	Harrison et al, 1996	USA	27.0	F	L	4	VSD	CC/DSA	C	I
21	Otto et al, 1998	Germany	28.0	F	R	2/3	None	DSA/ECHO	C	I
22	Heyneman et al, 2001	USA	25.0	F	R	1/2	PDA	CT/CC/PA	S	I
23	Heyneman et al, 2001	USA	31.0	F	R	1/2	VSD	CT/MRI/CC/PA	S	NA
24	Heyneman et al, 2001	USA	43.0	F	R	1/3	PH	CT/CC/PA	S	I

25	Pourmoghadam et al, 2003	USA	12.0	M	R	1/3	None	CC	C	W
26	Pourmoghadam et al, 2003	USA	9.0	F	R	1/3	None	MRI	C	W
27	Pourmoghadam et al, 2003	USA	13.0	M	R	1/3	None	CT/MRI/CC/PA	S	I
28	Ussia et al, 2004	Italy	5.0	F	R	1	None	CC	S	I
29	Tissot et al, 2008	USA	0.7	M	L	1	None	CC	C	I
30	Lee et al, 2008	China	0.1	M	R	3	ASD/MA/SV/PH	Chest CT/CC/PA	S	D
31	Mataciunas et al, 2009	Lithuania	12.0	M	R	1/3	None	CTA/CC/PA	C	NA
32	Argueta-Morales et al, 2009	USA	12.0	M	R	1/3	None	ECHO/CC/MRI	C	I
33	Gasparetto et al, 2010	Brazil	13.0	F	L	0	None	MRA	C	R

34	Cheng et al, 2010	China	2.0	M	R	1	None	Chest CT/CTA-3D/VQ scan	NA	NA
35	Kim et al, 2011	Korea	23.0	M	R	4	None	MDCT	C	I
36	Kim et al, 2011	Korea	39.0	F	R	0	None	MDCT	C	R
37	Dixit et al, 2011	India	7.0	F	R	1/2	None	CTA	C	NA
38	Dixit et al, 2011	India	3.0	F	R	2	None	CTA	C	NA
39	Kozak et al, 2011	Brazil	7.0	F	L	1/3	AVSD	CC/MSCT	NA	NA
40	Wang et al, 2012	China	18.0	M	L	0	None	CTA/ECHO	C	R
41	Savaş Bozbaş et al, 2012	Turkey	20.0	F	R	3	None	Chest CT/CC/PA	C	I
42	Cao et al, 2013	China	18.0	M	L	1/2	ASD/CTS/PH	CTA/CC/PA	NA	NA
43	Shen et al, 2013	China	8.0	F	R	1/2	PFO	MSCT	C	NA
44	Li et al, 2014	China	3.9	M	L	1/3	CECD/PH	Chest CT/Cardiac CECT	S	NA
45	Kong et al, 2014	China	40.0	M	L	1/2/3	PH	CTA	C	W

46	DeMastes-Crabtree et al, 2015	USA	12.0	F	R	1/2	None	CC/PA	S	I
47	Gómez Hernández et al, 2015	Spain	19.0	F	R	1/2	None	CC/PA	S	I
48	Liu et al, 2015	China	4.0	F	L	1/2	None	CTA/DSA	NA	NA
49	Tutar et al, 2016	Turkey	22.0	F	L	3	CTS	CECT	NA	NA
50	Narayanan et al, 2016	India	20.0	F	R	1/3	None	CECT	C	NA
51	Aparci et al, 2016	Turkey	19.0	M	L	3	CTS	CTA	S	NA
52	Kuronuma et al, 2017	Japan	23.0	F	R	0	None	CTA	C	NA
53	Patil et al, 2017	India	2.0	F	R	1/3	PH	CTA	C	W
54	Chen et al, 2017	China	0.4	M	L	1/3	PDA/PFO/PVRA/PH	CTA/ECHO	C	D
55	Chen et al, 2018	China	1.7	M	L	1/2	ASD/PH	AOG/Cardiac CT/ECHO/CC/PA	S	I

56	Chen et al, 2018	China	1.5	F	L	1/2	VSD/PH	CSE	S	I
57	Chen et al, 2018	China	1.6	M	L	1	PAS/PVRA/SA/SV	CC/Cardiac CT	S	NA
58	Chen et al, 2018	China	1.8	F	R	1/2	None	Chest CT/Cardiac CT/ECHO	C	W
59	Zhang et al, 2018	China	8.0	M	R	0	None	Chest CT/CTA	C	R
60	Zhang et al, 2018	China	2.0	M	R	1	None	CTA/ECT	C	I
61	Tang et al, 2018	China	2.9	M	L	2	None	CTA-3D	C	I
62	Dell'Amore et al, 2018	Italy	28.0	M	R	1/2	None	Angio-CT/CC/PA	S	I
63	Biradar et al, 2018	India	2.0	M	L	1	ASD	CTA	C	I
64	Guo et al, 2019	China	2.0	M	R	1	None	Chest CT/CTA/VQ scan	C	I
65	Guo et al, 2019	China	8.0	M	R	1	None	Chest CT/CTA	C	NA
66	Levin et al, 2019	USA	4.0	F	R	1/2	None	Chest CT/PET-CT/VQ scan	C	I

67	Levin et al, 2019	USA	0.5	M	R	0	None	CC/DSA	S	NA
68	Wong et al, 2020	Malaysia	4.0	F	R	2	None	CTA	C	I
69	Har-Even Cohn, et al, 2020	Canada	11.0	F	L	1/2/3	None	CTA	S	I
70	Har-Even Cohn, et al, 2020	Canada	3.0	M	L	1	PDA	CTA/ECHO	C	I
71	Har-Even Cohn, et al, 2020	Canada	13.0	F	L	0	None	CTA	C	R
72	Chiu et al, 2020	USA	0.1	NA	L	0	None	CTA/ECHO	S	R
73	Kromrey et al, 2020	Japan	6.0	M	R	4	None	CTA/DSA	NA	NA
74	Yang et al, 2022	China	3.0	M	L	1/2	None	CTA/CC/PA	C	I
75	Yang et al, 2022	China	12.0	M	R	1/2	None	CTA	C	W
76	Cong et al, 2022	Vietnam	19.0	M	L	1/2	None	Chest CT/CECT	C	I

77	Wang et al, 2022	China	30.0	F	R	3	PH	CECT/DSA	C	NA
78	Sim et al, 2022	Korea	62.0	F	R	1/2/3	None	CECT	C	NA
79	Weldetsadik et al, 2023	Ethiopia	13.0	M	L	1/2/3	None	CECT	S	I
80	This study	China	3.2	F	R	1/3	None	CTA	C	I
81	This study	China	4.9	F	R	1/2	None	CTA/DSA	C	I
82	This study	China	11.0	F	L	1/2	MVSA	CTA/DSA	C	I
83	This study	China	3.0	F	L	1/3	AVSD/CECD/PAS/PH	CTA	C	Lost to follow-up
84	This study	China	1.0	M	R	1/2/3	None	CTA/DSA	S	I
85	This study	China	5.2	M	R	1	None	CTA	C	I

Abbreviations:

Sex: F: Female; M: Male

Side: L: Left; R: Right

Main symptoms: 0: Asymptomatic; 1: Recurrent pneumonia; 2: Recurrent hemoptysis; 3: Exercise intolerance; 4: Others

Cardiac diseases: ASD: Atrial septal defect; AVSD: Atrioventricular septal defect; CECD: Complete endocardial cushion defect; CTS: Cor triatriatum sinistrum; MA: Mitral atresia; PAS: Pulmonary artery stenosis; PDA: Patent ductus arteriosus; PFO: Patent foramen ovale; PH: Pulmonary hypertension; PVRA: Pulmonary venous return anomaly; SA: Single atrium; SV: Single ventricle; MVSA: Membranous ventricular septal aneurysm; VSD: Ventricular septal defect

Diagnostic ways: Angio-CT: Angiography computed tomography; AOG: Aortography; CC: Cardiac catheterization; CECT: Contrast enhanced computed tomography; CSE: Cardiac surgery exploration; CT: Computed tomography; CTA: Computed tomography angiography; CT-3D: Computed tomography with 3-dimensional reconstruction; DSA: Digital subtraction angiography; ECHO: Echocardiography; ECT: Lung emission computed tomography; MDCT: Multidetector computed tomography; MRA: Magnetic resonance angiography; MRI: Magnetic resonance imaging; MSCT: Multislice computed tomography; PA: Pulmonary arteriography; PET-CT: Positron emission tomography–computed tomography; VQ scan: Ventilation-Perfusion scan

Therapeutic plans: C: Conservative treatment; S: Surgical treatment

Follow-up: D: Deceased; I: Symptoms improved; R: Remained asymptomatic; W: Symptoms worsened

Supplementary Table S2. Comparison of clinical features between Chinese UPVA patients and all reported UPVA patients worldwide.

	Chinese UPVA	Total UPVA	p value
**Variables**	n=27	n=85	
Age at diagnosis, years	3.2 (1.8, 8.0)	5.2 (2.0, 13.0)	NS
Sex, male/female	2/1	1/1	NS
**UPVA side, n (%)**	n=27	n=85	
Left	13 (48.1%)	35 (41.2%)	NS
Right	14 (51.9%)	50 (58.9%)	NS
**Clinical symptoms, n (%)**	n=25	n=77	
Asymptomatic	2 (8.0%)	8 (10.4%)	NS
Recurrent pneumonia	22 (88.0%)	61 (79.2%)	NS
Recurrent hemoptysis	13 (52.0%)	37 (48.1%)	NS
Exercise intolerance	8 (32.0%)	27 (35.1%)	NS
Others	0 (0%)	3 (3.9%)	NS
**Cardiac diseases, n (%)**	n=27	n=85	
None	15 (55.6%)	56 (65.9%)	NS
ASD	4 (14.8%)	7 (8.2%)	NS
VSD	2 (7.4%)	8 (9.4%)	NS
PDA	1 (3.7%)	8 (9.4%)	NS
MA	1 (3.7%)	1 (1.2%)	NS
SV	2 (7.4%)	2 (2.4%)	NS
PFO	2 (7.4%)	2 (2.4%)	NS

CECD	2 (7.4%)	2 (2.4%)	NS
CTS	1 (3.7%)	3 (3.5%)	NS
PVRA	2 (7.4%)	2 (2.4%)	NS
PAS	2 (7.4%)	2 (2.4%)	NS
SA	1 (3.7%)	1 (1.2%)	NS
MVSA	1 (3.7%)	1 (1.2%)	NS
PH	9 (33.3%)	17 (20.0%)	NS
**Therapeutic plan, n (%)**	n=24	n=78	
Conservative treatment	18 (75.0%)	52 (66.7%)	NS
Surgical treatment	6 (25.0%)	26 (33.3%)	NS
**Outcomes, n (%)**	n=18	n=56	
Remained asymptomatic	2 (11.1%)	6 (10.7%)	NS
Symptoms improved	11 (61.1%)	39 (69.6%)	NS
Symptoms worsened	3 (16.7%)	6 (10.7%)	NS
Deceased	2 (11.1%)	5 (8.9%)	NS

Abbreviations: ASD: Atrial septal defect; CECD: Complete endocardial cushion defect; CTS: Cor triatriatum sinistrum; MA: Mitral atresia; MVSA: Membranous ventricular septal aneurysm; NS: Not significant; PAS: Pulmonary artery stenosis; PDA: Patent ductus arteriosus; PFO: Patent foramen ovale; PH: Pulmonary hypertension; PVRA: Pulmonary venous return anomaly; SA: Single atrium; SV: Single ventricle; VSD: Ventricular septal defect
